# Supplementary figures and images for: Post-Vaccination Neutralization Responses to Omicron Sub-Variants
Source: Vaccines (Basel). 2022 Oct 20;10(10):1757. doi: 10.3390/vaccines10101757 (PMC9607453; doi:10.3390/vaccines10101757)

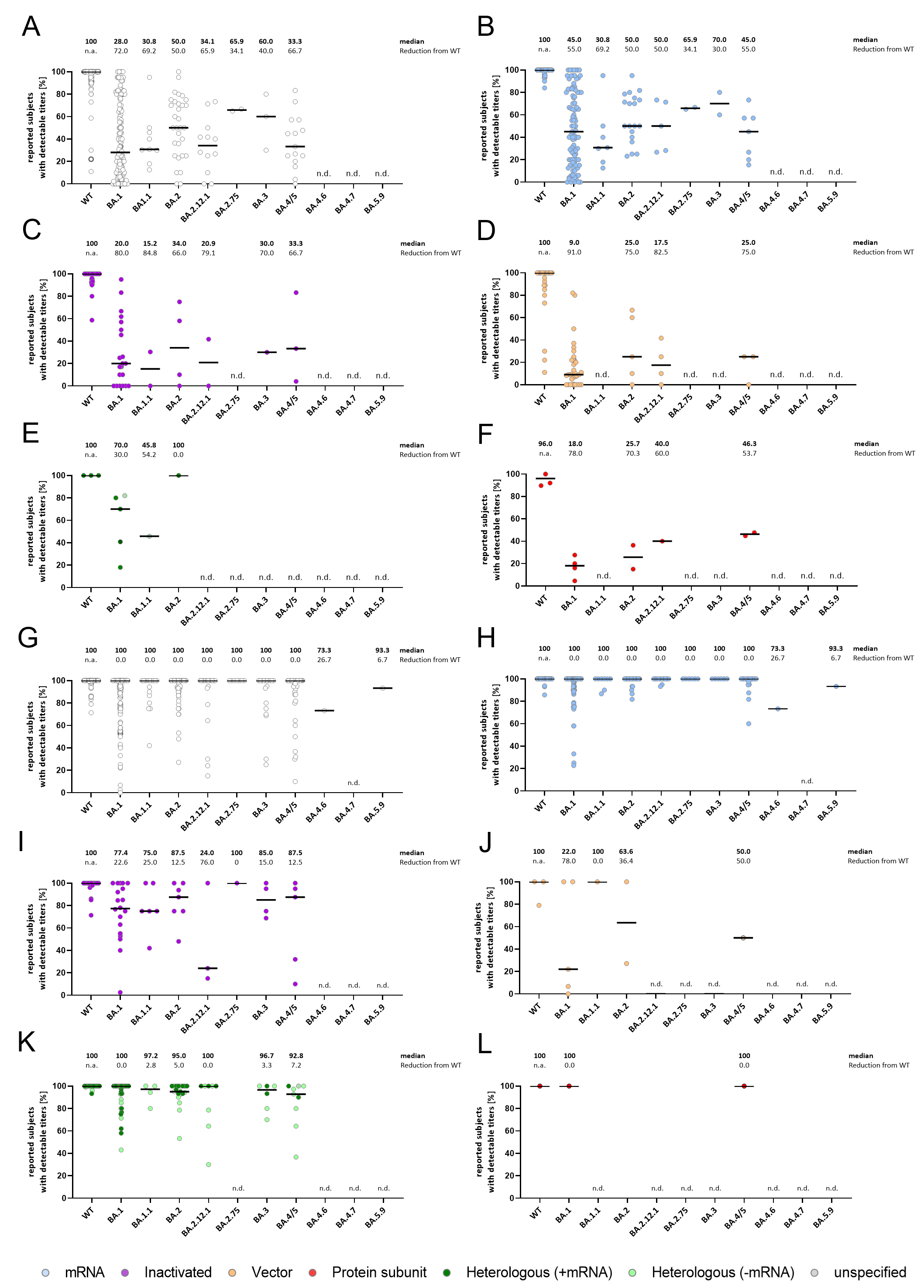

Supplement: Supplementary file 1 [file vaccines-10-01757-s001.zip › Supplementary_figure_S1.png]

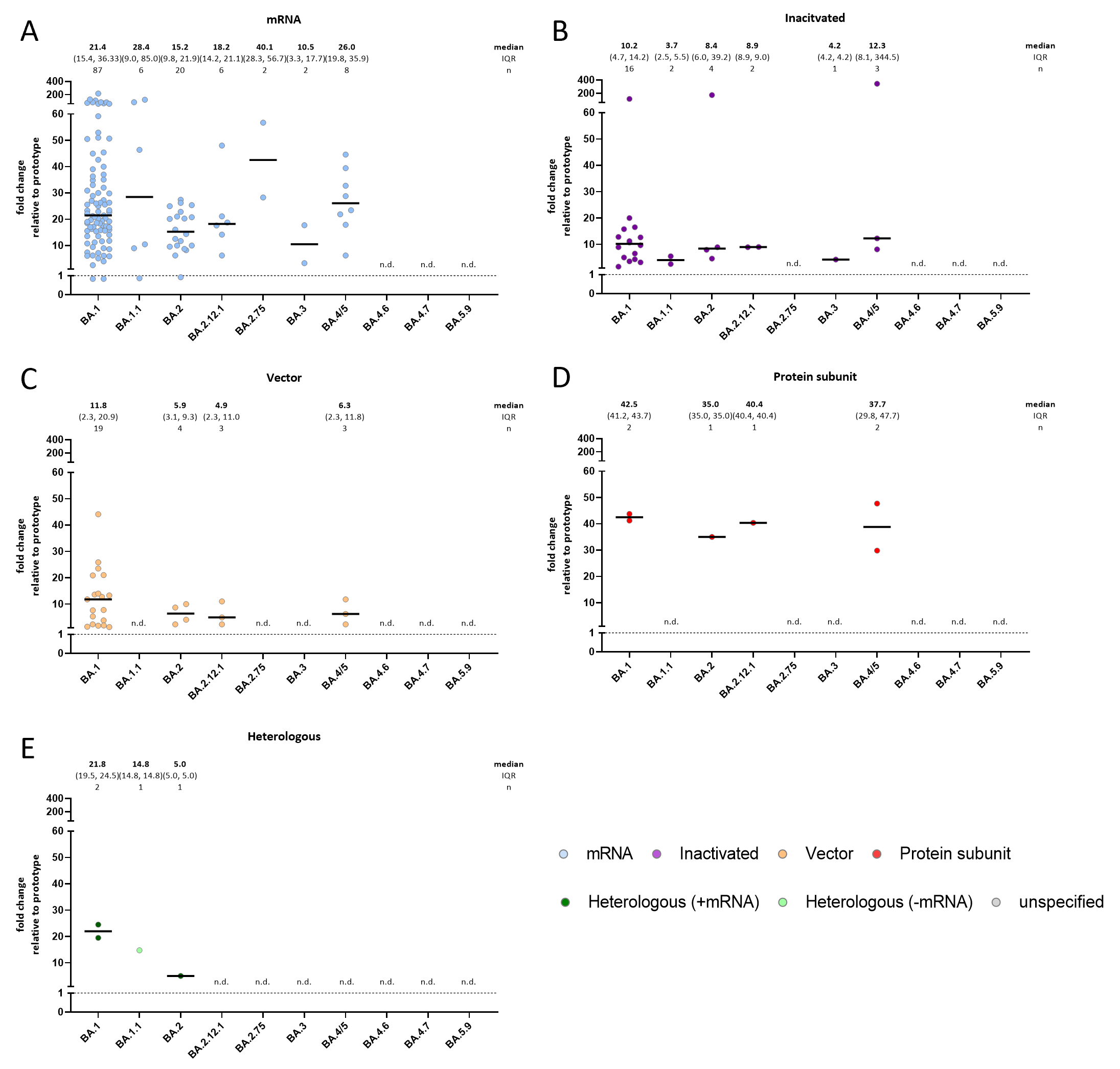

Supplement: Supplementary file 1 [file vaccines-10-01757-s001.zip › Supplementary_figure_S2.png]

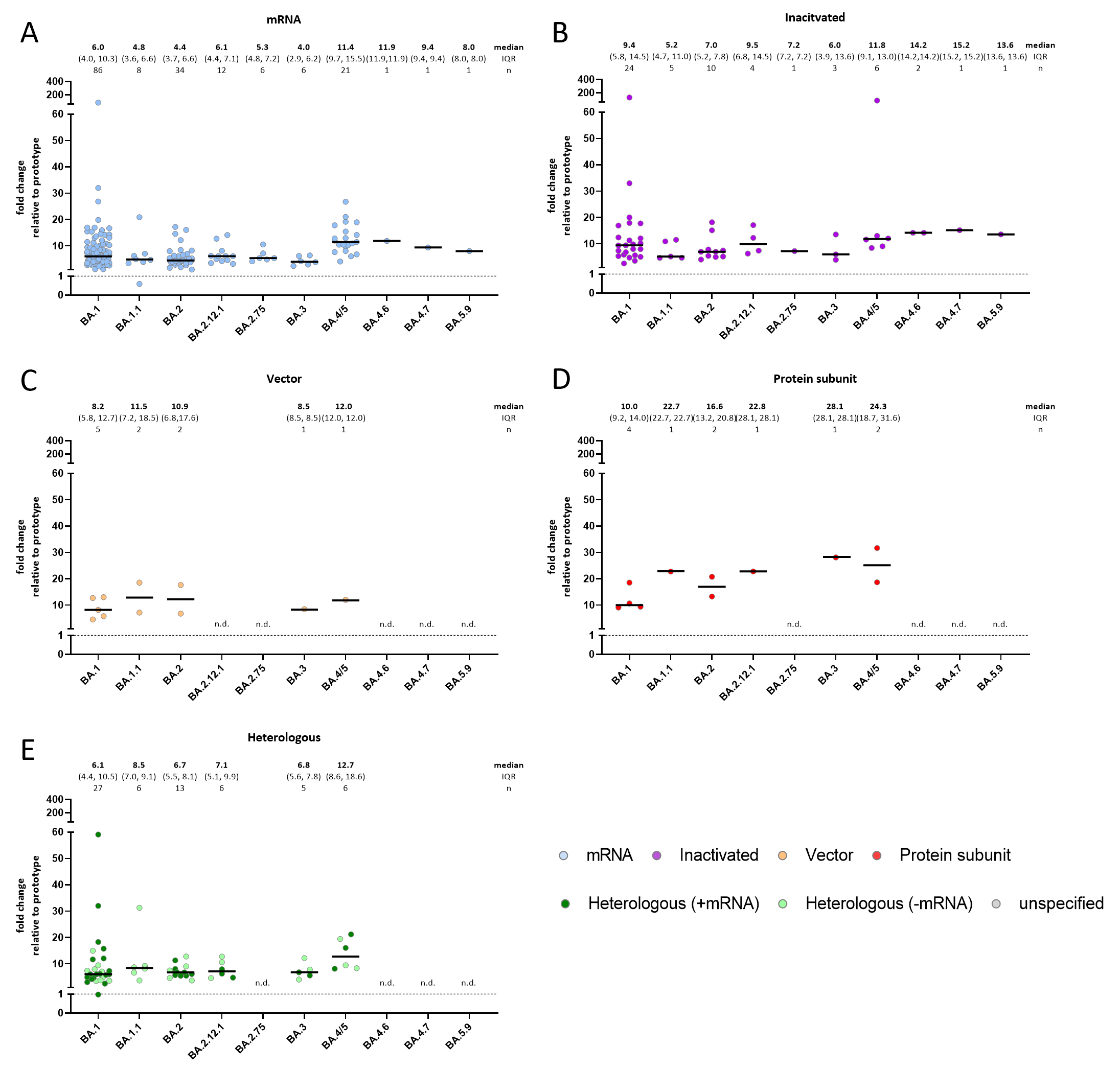

Supplement: Supplementary file 1 [file vaccines-10-01757-s001.zip › Supplementary_figure_S3.png]
